# Self and proxy symptom reporting in glioma patient-caregiver dyads: the role of psychosocial function in rating accuracy

**DOI:** 10.1186/s41687-024-00726-8

**Published:** 2024-07-17

**Authors:** Meagan Whisenant, Stella Snyder, Shiao-Pei Weathers, Eduardo Bruera, Kathrin Milbury

**Affiliations:** 1 Department of Behavioral Science, The University of Texas MD Anderson Cancer Center, Houston, TX USA; 2 Department of Psychology, School of Science, Indiana University-Purdue University at Indianapolis, Indianapolis, IN USA; 3 Department of Neuro-Oncology, The University of Texas MD Anderson Cancer Center, Houston, TX USA; 4 Department of Palliative, Rehabilitation and Integrative Medicine, The University of Texas MD Anderson Cancer Center, Houston, TX USA

**Keywords:** Primary brain tumors, Informal caregivers, Family caregivers, Illness communication, Depressive symptoms, Quality of life

## Abstract

**Background:**

Illness-related communication and depressive symptoms within families may play an important role in caregivers’ ability to accurately understand patients’ symptom burden. We examined the associations between these psychosocial factors and symptom accuracy in patients with glioma and their family caregivers.

**Methods:**

Patients and caregivers (*n* = 67 dyads) completed measures of depressive symptoms (CES-D), illness communication (CICS), and QOL (SF-36). Patients reported on their own cancer-related symptoms (MDASI-BT) while caregivers reported on their perception of the patients’ symptoms (i.e., proxy reporting). Paired *t*-tests and difference scores were used to test for agreement (absolute value of difference scores between patients and caregiver proxy symptom and interference severity reports) and accuracy (caregiver underestimation, overestimation, or accurate estimation of patient symptom and interference severity).

**Results:**

Clinically significant *disagreement* was found for all means scores of the MDASI-BT subscales except for gastrointestinal symptoms and general symptoms. Among caregivers, 22% overestimated overall symptom severity and 32% overestimated overall symptom interference. In addition, 13% of caregivers underestimated overall symptom severity and 21% of caregivers underestimated overall symptom interference. Patient illness communication was associated with agreement of overall symptom severity (*r*=−0.27, *p* = 0.03) and affective symptom subscale (*r*=−0.34, *p* < 0.01). Caregivers’ reporting of illness communication (*r*=−0.33, *p* < 0.01) and depressive symptoms (*r* = 0.46, *p* < 0.0001) were associated with agreement of symptom interference. Caregiver underestimating symptom severity was associated with lower patient physical QOL (*p* < 0.01); caregiver underestimating symptom interference was associated with lower patient physical QOL (*p* < 0.0001) and overestimating symptom interference was associated with lower patient physical QOL (*p* < 0.05). Patient and caregiver mental QOL was associated with caregiver underestimating (*p* < 0.05) and overestimating (*p* < 0.05) symptom severity.

**Conclusion:**

The psychosocial context of the family plays an important role in the accuracy of symptom understanding. Inaccurately understanding patients experience is related to poor QOL for both patients and caregivers, pointing to important targets for symptom management interventions that involve family caregivers.

## Introduction

Considering the high disease burden, symptom management for patients with glioma (the majority of primary malignant brain tumors) is an important aspect of successful oncological care [1]. While some symptoms, including depression, fatigue and sleep disturbances, are commonly experienced by cancer patients regardless of disease site and treatment type, other symptoms, particularly in the cognitive and neurological domains, tend to be brain tumor specific [2, 3]. In addition to functional declines, mood and personality changes present challenges for patients with glioma and their families alike [4, 5]. In fact, as the disease progresses, patients with glioma heavily rely on family members, particularly their spouses/romantic partners, for care and support [6–8].

Open illness-related communication within families, meaning members’ willingness to discuss the patient’s illness, may play an important role in understanding the patient’s symptom experience. Yet, families often do not discuss the patient’s symptoms or disclose their concerns and feelings to each other for several different reasons. For instance, Zhang and colleagues interviewed caregivers of cancer patients to evaluate their reasons for not engaging in open communication with the patient [9]. Most of the families in this qualitative study avoided communication because they did not want to distress the patient, with the intention to protect their loved one (i.e., protective buffering), which is consistent with other cancer literature [10]. Other reasons included the desire for mutual protection against harmful words or actions and maintaining optimism [9]. However, attempts to minimize discussion of distressing topics are associated with increased symptoms of anxiety and depression and may lead to decreased relationship satisfaction and emotional distancing within families experiencing cancer [11, 12].

Without open illness communication, caregivers of patients with cancer and other chronic disease rely on their *perceptions* of the patient’s symptom burden to guide the type of support and care they provide to the patient [13, 14]. In a longitudinal study, Silveira and colleagues examined the association between changes in caregiver accuracy in their perceptions of the glioma patient’s symptom severity and changes in actual patient symptom outcomes. Accuracy was defined by the difference scores the patient and caregiver reporting. Overall, their findings suggest that caregivers overestimate the severity of patients’ symptoms, and unfortunately, their accuracy decreases over time (i.e., difference score became larger) [13]. In fact, recent findings involving patients with high-grade glioma suggest that caregivers’ proxy ratings of patients’ symptom severity and quality of life (QOL) shows generally low agreement as defined by concordance correlation coefficient, particularly if patients reveal neurocognitive impairments [15]. In the broader advanced cancer literature, caregivers more often overestimate patient symptoms, especially when reporting on psychological symptoms [16]. The role of illness communication in the dyad’s agreement of symptom ratings is yet to be explored.

Moreover, while previous research has mainly focused on identifying patients’ clinical variables (e.g., performance status, neurocognitive function) that may be related to symptom agreement, there is reason to believe that caregivers’ psychosocial characteristics may play an important role in their ability to accurately understand patients’ symptom experience [13–15]. Depression may be a key characteristic as it is related to cognitive biases that influence individuals’ perceptions of themselves and their surroundings. One of the most pervasive tendencies of depressed individuals is to focus on negative stimuli (negative attentional bias), and thus, depressed caregivers may overestimate patients’ symptom experience [17]. Alternatively, because depression is associated with an increased self-focus, depressed caregivers may be too distracted by their own experience to accurately interpret and pay attention to the patient’s symptoms.

Thus, accurately interpreting patients’ symptom experiences may facilitate caregivers’ appropriate level of care and supportive behaviors, and thus increase patient QOL. In comparison to overestimating symptoms, an accurate understanding may decrease unnecessarily burdening the caregiver, and thus, protect caregiver QOL. The current study seeks to extend the literature by examining the associations between symptom accuracy and patient and caregiver QOL, as previous research has primarily focused on examining the validity of proxy reporting as the main study goal. We also seek to gain a better understanding of the role of patients’ and caregivers’ psychosocial functioning in symptom understanding (i.e., accurate ratings) to inform the development of family-focused symptom management interventions. For this study, similar to Silveira et al., we define accuracy in terms of difference scores between patient and caregiver reports on patient symptom severity and interference [13]. Using a 1-point rating difference (which is validated as a clinically meaningful difference), we seek to determine underestimation (a difference score of ≥ 1), overestimation (a difference score of ≤ −1), or accuracy (difference score > −1 and < 1) in symptom severity and interference.

The primary goals of this exploratory study were to examine:

1. The accuracy in symptom reporting in patients and their caregivers using the MD Anderson Symptom Inventory-Brain Tumor Module (MDASI-BT).
2. The association between both patient and caregiver illness communication and depressive symptoms and MDASI-BT symptom accuracy ratings.
3. The extent to which accuracy in ratings is associated with patient and caregiver physical and mental QOL.

## Methods

This secondary analysis reports on cross-sectional measures that were collected prior to randomization as part of the baseline assessment of a feasibility randomized controlled trial seeking to pilot-test a yoga intervention as a supportive care strategy for patients with glioma and their family caregivers (NCT02481349) [18]. This study was approved by the Institutional Review Board at The University of Texas MD Anderson Cancer Center.

### Participants

To be eligible for the parent study, patients had to (1) be newly diagnosed with a glioma and planning to receive at least 4 weeks of radiotherapy with at least 20 fractions; (2) have a Karnofsky Performance Status (KPS) of 80 or above; and (3) have a family caregiver (e.g., spouse/partner, adult child, sibling) who is willing to participate. Patients and caregivers had to be at least 18 years old, be able to provide informed consent, and be able to read and speak English. Patients with cognitive deficits that would hinder the completion of the questionnaires were excluded from the study.

### Procedures

Research staff identified potential participants via the Institution’s computerized appointment system, approached potential participants during their initial radiotherapy consult or simulation visit, screened patients and caregivers for eligibility, and asked them for informed consent. If a patient’s caregiver was not present during the initial contact, the patient was asked for permission to contact the caregiver via telephone to obtain consent. Once consent was obtained, patients and caregivers were asked to complete electronically administered survey measures independently via REDCap. Patients and caregivers within dyads completed study measures within 48 h of each other. Each participant received a $20 gift card after completing this survey.

### Measures

*Demographics* were provided by participants, including age, gender, race/ethnicity, employment status, education and type of relationship with the family caregiver (e.g., spouse, adult child).

*Medical factors* including diagnosis (i.e., grade, tumor location, lateralization, time since diagnosis), prior surgery, concurrent chemotherapy, seizure history, and steroid and anticonvulsant treatment were extracted from patients’ electronic health records (EHR).

*Patient Cancer-Specific Symptoms* were assessed with the MD Anderson Symptom Inventory-Brain Tumor module (MDASI-BT), which consists of 13 core items and 9 brain tumor-specific items assessing symptom severity, and 6 items assessing interference with daily life on a scale from 0 to 10 [19]. The MDASI-BT measures the severity of six underlying constructs including affective (distress, fatigue, sleep disturbance, sadness, and irritability), cognitive (difficulty understanding, difficulty remembering, difficulty speaking, and difficulty concentrating), neurologic deficit (seizures, numbness, pain, and weakness), treatment-related (dry mouth, drowsiness, and lack of appetite), generalized symptom (change in appearance, change in vision, change in bowel patterns, and shortness of breath), and a gastrointestinal-related factor (nausea and vomiting). We present these subscales along with the total symptom severity and interference (symptom-related interference with general activity, mood, work, relations with other people, walking, and enjoyment of life) subscales. Patients rated their own symptoms. Caregivers were instructed to rate their perception of the patient’s symptoms (i.e., proxy ratings).

#### Psychosocial factors

Depressive symptoms were assessed with the Center for Epidemiologic Studies Depression Scale (CES-D), a 20-item self-report measure of 20 items focusing on the affective component of depression (Cronbach’s alpha = 0.86). A score of ≥ 16 is considered the cut-off to screen for a depressive disorder [20]. Illness Communication was assessed with the Couples Illness Communication Scale (CICS), a 4-item instrument capturing illness-related communication regarding patient and caregiver comfort with discussing cancer [21]. Each item is rated on a 5-point Likert scale ranging from 1 (Strongly Disagree) to 5 (Strongly Agree). Higher scores denote better illness communication.

*Overall QOL* was measured with the Medical Outcomes Study 36-item short-form survey (SF-36) which assessed 8 distinct domains: physical functioning, physical impediments to role functioning, pain, general health perceptions, vitality, social functioning, emotional impediments to role functioning, and mental health yielding a mental and physical composite summary (MCS and PCS, respectively).

### Data analysis

Descriptive statistics (e.g., frequencies, ranges, means, and standard deviations) were calculated for all measures. Paired *t*-tests were used to examine group differences (i.e., patient vs. caregiver) for each study measure along with inter-correlations coefficients (ICC) were used to test for interdependence in patient and caregiver scores. These ICCs are provided to show dyadic interdependence in scores rather than congruence ratings. Similar to Silveira’s approach, to examine accuracy scores, we trichotomized the difference scores (patient score − caregiver proxy score) in the following manner: a difference score of ≤ −1 (more negative values) denotes clinically significant overestimating; a difference score of ≥ 1 denotes clinically significant underestimating; and difference scores between > −1 and < 1 were considered accurate (i.e., not clinically significant different) [13]. We selected 1-point difference as it corresponds with the clinically minimum difference scores reported in MDASI validation studies (0.5 SD) and is universally used for clinically significant difference in previous studies using the MDASI [22]. To examine the association between psychosocial variables (illness communication and depressive symptoms) and symptom accuracy on the overall symptom severity and symptom interference subscales, Pearson bivariate correlations were examined separately for patients and caregivers. Please note that for interpretation purposes of these associations, we converted the difference score assessing accuracy into absolute values. Higher absolute values indicate lower agreement. To tests for the dyadic level associations between symptom agreement and QOL (i.e., PCS and MCS scores) multi-level modeling (MLM) was used with the PROC MIXED procedure in SAS (Version 9.4, SAS, Cary, NC, USA) accounting for the nested data structure (i.e., individuals within dyads). We tested the main effect of accuracy and role (patient or caregiver), as well as the interaction between accuracy and role (i.e., role × accuracy). Significant main effects for accuracy and interaction effects for role × accuracy were followed with Bonferroni posthoc comparison analyses to correct for multiple comparisons.

## Results

### Sample description

Patient and caregiver (*n* = 67 dyads) demographics and patient medical factors are portrayed in Table 1. Briefly, patients had a mean age of 46.8 years (SD = 14.4 years), 63% of patients were male, 92.5% were non-Hispanic White, and 73.2% had a college or more advanced degree. The majority of patients were staged at Grade III–IV (83.6%) with a mean length of diagnosis of 18 weeks (range = 1.4 weeks to 4.2 years). Caregivers had a mean age of 50.9 years (SD = 11.9 years), 79% were female, 69% were spouses, 89.5% were non-Hispanic White, 73.2% had a college or more advanced degree, and 50.8% were employed. Regarding depressive symptoms, 39.1% of patients and 48.6% of caregivers endorsed levels of depression consistent with clinical depression (CES-D scores, patient: mean = 14.51, SD = 8.60; caregiver: mean = 15.57, SD = 9.67, paired *t* =−0.74, *p* = 0.46).

**Table 1:** Sample demographic and medical characteristics (*n* = 67)

Characteristic	Patients	Caregivers
Age, mean ± SD (range), years	46.8 ± 14.4 (18–75)	50.9 ± 11.9 (28–83)
Gender, *n* (%)		
Male	42 (62.7)	14 (20.9)
Female	25 (37.3)	53 (79.1)
Race, *n* (%)		
White	62 (92.5)	60 (89.5)
Asian	2 (3.0)	3 (4.5)
More than one race	2 (3.0)	2 (3.0)
Declined to answer	1 (1.5)	2 (3.0)
Hispanic, *n* (%)	9 (13.4)	9 (13.4)
Married, *n* (%)	50 (74.6)	56 (83.6)
Education level, *n* (%)		
High school graduate	8 (11.9)	4 (6.0)
Some college or technical school	10 (14.9)	13 (19.4)
College graduate	23 (34.4)	31 (46.3)
Graduate school	26 (38.8)	18 (26.9)
Unknown	0 (0.0)	1 (1.5)
Employment status, *n* (%)		
Employed full- or part-time	25 (37.3)	34 (50.8)
Taking time off for treatment	20 (29.9)	11 (16.4)
Retired	8 (11.9)	9 (13.4)
Other (e.g., unemployed, on disability)	11 (16.5)	11 (16.5)
Unknown	3 (4.5)	2 (3.0)
Household income, *n* (%)		
≤$50,000	7 (10.5)	5 (7.5)
$50,001–$75,000	8 (11.9)	6 (9.0)
$75,001–$100,000	11 (16.4)	9 (13.4)
>$100,000	28 (41.8)	41 (61.2)
Unknown	13 (19.4)	6 (9.0)
Caregiver type, *n* (%)		
Spouse		46 (68.7)
Other		21 (31.3)
Disease grade, *n* (%)		
Low grade (I and II)	9 (13.4)	
High grade (III and IV)	56 (83.6)	
Unknown	2 (3.0)	
Time since diagnosis, years, mean ± SD (range)	0.35 ± 0.71 (0.03–4.2)	
Tumor location, *n* (%)		
Frontal	34 (50.7)	
Temporal	16 (23.9)	
Other	17 (25.4)	
Lateralization of tumor, *n* (%)		
Left	25 (37.3)	
Right	33 (49.3)	
Bi-lateral	7 (10.4)	
Unknown	2 (3.0)	
Concurrent treatments, *n* (%)		
Surgery	61 (91.0)	
Chemotherapy	64 (95.5)	
Steroid medication	27 (40.3)	
Anti-convulsant	55 (82.1)	
History of seizures, *n* (%)	38 (56.7)	
Functional status (KPS), *n* (%)		
Normal no complaints, no evidence of disease	12 (18.2)	
Able to carry on normal activity; minor signs or symptoms of disease	43 (65.2)	
Normal activity with effort; some signs or symptoms of disease	11 (16.7)	
Unknown	1 (1.5)	

*Note KPS* Karnofsky Performance Status

### Accuracy of self and proxy symptom ratings

Based on paired *t*-test analyses, patient and caregiver proxy ratings did not statistically significantly differ on any of MDASI-BT subscales at *p* < 0.05. ICCs of patient and caregiver proxy ratings were statistically significant (except of the cognitive and GI subscales) ranging from small to moderate coefficients (0.09 − 0.50) (Table 2). Regarding accuracy, 22% of caregivers overestimated and 13% underestimated overall symptom severity and 32% of caregivers overestimated and 21% underestimated symptom interference. Table 3 presents detailed accuracy results including the absolute value of mean difference scores to demonstrate the magnitude of disagreement (regardless of the direction of inaccuracy). Clinically significant inaccuracy (means scores > 1.0) were found for all means scores of the MDASI-BT subscales except for GI symptoms and general symptoms.

**Table 2:** Patient self-ratings and caregiver proxy ratings MDASI-BT subscales, psychosocial function and quality of life

Variables	Patient mean (SD)	Caregiver mean (SD)	ICC	Paired *t*-test
MDASI-BT (*n =* 67)				
Affective	2.77 (2.25)	3.17 (2.02)	0.32**	−1.32
Cognitive	1.52 (1.90)	2.08 (2.00)	0.09	−1.73
Neurologic	1.34 (1.75)	1.69 (1.65)	0.50***	−1.70
Gastrointestinal	0.63 (1.17)	0.63 (1.02)	0.20	−0.04
General disease	1.11 (1.31)	1.15 (1.05)	0.47***	−0.28
Treatment-related	1.67 (1.73)	1.92 (1.93)	0.32**	−0.95
Overall symptoms	1.64 (1.34)	1.93 (1.27)	0.26*	−1.52
Interference	2.56 (2.44)	3.21 (2.42)	0.31*	−1.86
Depressive symptoms	14.69 (8.52)	15.69 (9.67)	0.28*	−0.74
Illness communication (*n* = 64)	16.31 (3.18)	14.59 (3.94)	−0.03	2.68*
Physical composite summary (*n* = 65)	35.99 (6.59)	44.17 (6.90)	0.28*	−8.14***
Mental composite summary (*n* = 65)	45.67 (9.14)	44.40 (11.14)	0.14	0.76

*Note MDASI-BT* MD Anderson Symptom Inventory-Brain Tumor, *ICC* Intraclass correlation coefficient

*p-value < 0.05; **p-value < 0.01; ***p-value < 0.001

**Table 3:** Patient and caregiver agreement (*n* = 67)

MDASI-BT	Difference mean score (SD) Range	Accurate n (%)	Overestimated n (%)	Underestimated n (%)	Rank order^a^
Affective	1.93 (1.60)	23 (34.3)	27 (40.3)	17 (25.4)	6
	0–7.20				
Cognitive	1.73 (2.06)	29 (43.3)	26 (38.8)	12 (17.9)	5
	0–8.75				
Neurologic	1.14 (1.31)	34 (50.7)	22 (32.8)	11 (16.4)	3
	0–7.25				
Gastrointestinal	0.80 (1.13)	43 (64.2)	15 (22.4)	9 (13.4)	1
	0–5.00				
General disease	0.85 (0.90)	41 (82.1)	14 (20.9)	12 (17.9)	2
	0–4.75				
Treatment-related	1.54 (1.50)	28 (41.8)	21 (31.3)	18 (26.9)	4
	0–6.00				
Overall symptoms severity	1.14 (1.14)	40 (59.7)	17 (25.4)	10 (14.9)	
	0–4.86				
Interference	2.14 (1.99)	28 (41.2)	24 (35.3)	16 (23.5)	
	0–7.83				

*Note MDASI-BT* MD Anderson Symptom Inventory-Brain Tumor; the difference mean score is the absolute value of the difference scores (patient score − caregiver proxy score) so that higher values represent lower accuracy; Accurate, patient score − caregiver proxy score > −1 and < 1; Overestimated, patient score − caregiver proxy score ≤ −1 (more negative values); Underestimated, patient score − caregiver proxy score ≥ 1

^a^From highest to lowest in accuracy

### Bivariate correlates of symptom accuracy ratings and psychosocial variables for patients and caregivers

Patient illness communication was statistically significantly associated with agreement in overall symptom severity (*r*=−0.27, *p* = 0.03) and affective symptom subscale (*r*=−0.34, *p* < 0.01) so that the better the patient communication scores on the CICS, the lower the symptom inaccuracy. Patient depressive scores were not statistically significantly associated with overall symptom severity agreement (*r* = 0.16, *p* = 0.19). Patient illness communication (*r*=−0.05, *p* = 0.69) and depressive symptoms (*r* = 0.10, *p* = 0.41) were not statistically significantly associated with symptom interference agreement.

For caregivers, neither their illness communication (*r* = 0.16, *p* = 0.19) nor their depressive symptoms (*r*=−0.01, *p* = 0.95) were statistically significantly associated with symptom severity agreement. Yet, caregivers’ reporting of illness communication (*r*=−0.33, *p* < 0.01) and depressive symptoms (*r* = 0.46, *p* < 0.0001) were statistically significantly associated with symptom interference agreement so that those with better communication and lower depressive symptoms reported less disagreement on the symptom interference subscale.

### Dyadic level analyses on accuracy and quality of life

Regarding PCS scores, MLM analyses (accounted for the dyadic level data structure) revealed a statistically significant main effect for role (F = 65.15, *p* < 0.0001; means, patient = 34.38; caregiver = 43.48) and accuracy of symptom severity (F = 5.56, *p* = < 0.01; PCS means, accurate = 41.48, overestimated = 38.67, underestimated = 36.56) and a statistically significant interaction between accuracy and role (F = 3.01, *p* = 0.05; patient means, accurate = 37.63, overestimated = 35.96, underestimated = 29.56; caregiver means, accurate = 45.32, overestimated = 41.37, underestimated = 43.74). Based on post hoc comparisons, the PCS means were statistically significantly lower (i.e., worse physical QOL) for patients whose caregiver underestimated versus accurately perceived (*p* < 0.01) symptom severity. For caregivers, none of the comparisons were statistically significant.

We also revealed a statistically significant main effect for role (F = 59.52, *p* < 0.0001) and accuracy (F = 13.72, *p* < 0.0001; means, accurate = 42.82, overestimated = 39.68, underestimated = 35.68) but no role × accuracy interaction effect (F = 0.14, *p* = 0.86) for symptom interference. When caregivers accurately perceived symptom interference, both patients and caregivers reported statistically significantly higher PCS scores than when caregivers overestimated (*p* < 0.05) or underestimated (*p* < 0.0001) symptom interference.

Regarding MCS scores, while there was no statistically significant main effect for role (F = 0.01, *P* = 0.93; means, patient = 44.58; caregiver = 43.36), the main effect for accuracy of symptom severity was statistically significant (F = 4.38, *p* < 0.05; means, accurate = 45.73, overestimated = 46.94, underestimated = 39.23). The interaction between accuracy and role was not statistically significant (F = 1.33, *p* = 0.27). When caregivers underestimated symptom severity, regardless of role, participants reported statistically significantly lower MCS scores than when accurately perceiving (*p* < 0.05) or overestimating (*p* < 0.05) symptom severity.

We did not find a statistically significant main effect for role (F = 0.15, *p* = 0.70) or accuracy of symptom interference (F = 1.52, *p* = 0.22; means, accurate = 46.25, overestimated = 42.83, underestimated = 45.37), and the role × accuracy interaction effect was marginally statistically significant (F = 2.08, *p* = 0.06; patient means, accurate = 48.43, overestimated = 44.54, underestimated = 42.34; caregiver means, accurate = 44.06, overestimated = 41.04, underestimated = 48.15). None of the posthoc comparisons were statistically significant.

## Discussion

In this study, we assessed accuracy in symptoms reported among patients and caregiver proxy to further uncover symptom understanding in families coping with glioma. In addition, we examined the association between illness communication and depressive symptoms and accuracy, as well as the extent to which accuracy is associated with patient and caregiver QOL. Our findings suggest that there are clinically significant *inaccuracies* between patient and caregiver proxy rating; patient illness communication is associated with accuracy in symptom severity; and caregiver illness communication and depressive symptoms are associated with accuracy in symptom interference. We also revealed that accuracy in symptom ratings is associated with QOL. More specially, in comparison to accurately perceiving patient symptoms, underestimating symptom severity is significantly associated with poorer patient physical QOL and patients and caregiver mental QOL. Moreover, in comparison to accuracy, both under- and overestimating symptom interference is significantly associated with poorer patient and caregiver physical QOL.

Our accuracy findings shed further light on the existing literature that includes conflicting results regarding the degree of agreement in patient and proxy symptom and QOL [23–25]. While our findings did not suggest statistically significant mean differences between patient and caregiver proxy ratings of symptom severity and interference, we did find clinically meaningful inaccuracies, with 22% and 32% of caregivers overestimating and 13% and 21% of caregivers underestimating overall symptom severity and interference, respectively. Moreover, the overlap between patient and caregiver ratings as assessed by ICCs was only small to moderate. As such, our findings support a recent, large study of congruence between high-grade glioma patient and caregiver QOL reports suggesting little agreement on both generic and disease-specific outcomes [15]. In our sample, only the GI symptoms and general symptoms subscales were found to have high accuracy, suggesting that agreement in proxy reporting may differ among symptom domains, with greater inaccuracy found in symptoms associated with emotional and cognitive functioning as compared to physical functioning, which may be more easily observed by caregivers [15, 16]. In addition, within our sample, the patient mean score for the GI symptoms and general symptoms subscales were lower than other subscales, suggesting that accuracy may be higher for symptoms that patients experience at mild severity.

Of note, our findings extend the existing literature in an important manner. To the best of our knowledge, this is the first study to examine the association between family psychosocial factors (illness communication and depressive symptoms) and agreement in proxy symptom reporting. Here, we found that illness communication may influence accuracy, in particular patient illness communication may influence symptom severity accuracy (especially affective symptoms) and caregiver illness communication may impact symptom interference accuracy. Considering that accuracy was lowest for affective symptoms, the caregiver may not be aware of such symptoms unless the patient discloses concerns, especially in situations of patient buffering (e.g., concealing their concerns and feelings). Moreover, the second lowest accuracy was for symptom interference, again, suggesting that open communication may be necessary for caregivers to understand how symptoms interfere with patients’ daily life.

In addition, caregiver depressive symptoms may influence symptom interference accuracy. Inaccuracy in symptom interference ratings among dyads where the caregiver reports depressive symptoms may result from a reduced ability of the caregiver to perceive the patient’s symptom impacting daily life due to cognitive processes such as rumination, inattention, and a heightened self-focus, that is common among depressed individuals [26, 27]. Alternatively, in the presence of caregiver depressive symptoms, the patient may avoid or hide their own symptom-related interference seeking to minimize caregiver burden, maintain optimism, or avoid guilt. Considering that caregiver depressive symptoms are inversely associated with patient illness communication, both of these processes may be underlying mechanisms which should be further delineated in future studies to optimally inform targets of psychosocial interventions.

We also revealed that accurately perceiving the patient symptom experience is important beyond a measurement perspective, as accuracy is associated with patient and caregiver QOL. In situations where caregivers’ underestimate symptom severity, poor patient physical and mental QOL may result from inappropriate symptom management and lack of needed care and support. In contrast, overestimating symptom interference may result in poor caregiver physical QOL due to an inaccurate perception of the level of assistance the patient needs, possibly resulting in unnecessary physical burden. However, the links between symptom understanding (i.e., accuracy) and caregiving behaviors are speculative, and future research is needed to examine these associations, ideally in a prospective manner.

Moreover, although our findings suggest the importance of managing caregiver depressive symptoms and facilitating illness communication to mitigate inaccuracy and improve QOL, additional research is needed to better understand the mechanisms by which these constructs may lead to disagreement in patient and proxy symptom. More specifically, further work is needed to establish the contribution of sociodemographic characteristics, patient protective buffering, and caregiver’s behaviors including cognitive processes that interfere with accurately understanding the patient’s experience. Elucidating such mechanisms will inform the development of dyadic supportive care interventions aimed at mitigating symptoms in patients while reducing strain in caregivers. In addition, given that accuracy may be dependent on the caregivers’ depressive symptoms and illness communication in the family, clinicians should consider the psychosocial function of caregivers when requesting and interpreting proxy symptom reports.

This study has a few limitations. Importantly, the number of response options on the MDASI-BT may have influenced agreement. The MDASI-BT measure offers a scale ranging from 0 to 10, with 11 response options. It is possible that greater accuracy would be seen with a smaller response option scale, simply given the likelihood of selecting the same option with fewer choices. The research design was cross-sectional and therefore we cannot establish causality and directionality of associations. Patient cognitive functioning was not assessed in the parent study, therefore we were not able to examine the association between patient cognitive functioning and patient-proxy symptom reporting agreement. Further exploration is needed to understand how tumor location may influence patient perception and symptom reporting, and thus accuracy. We only assessed depressive symptoms; however, patient and caregiver anxiety symptoms may also be associated with symptom perception (e.g., due to associated hypervigilance that is associated with anxiety). Based on survey completion times, patients and caregivers provided symptom severity reports within 48 h of each other. This lag time could account for discrepancy in symptom severity reports, given the dynamic nature of symptom experiences. Our sample size is relatively small and included patients with high-performance status and a homogenous treatment history, as well as dyads that were generally highly educated and married. Consequently, it is unclear if our findings generalize to the larger population, including patients with lower performance across the treatment trajectory and families from diverse backgrounds.

Nonetheless, our findings provide insight into the accuracy of caregiver proxy reporting for symptom severity and interference in neuro-oncology setting. Although prior research has examined the relationship between patient neurocognitive functioning and patient-proxy agreement in symptom and QOL reporting, here we offer initial evidence of a link between illness communication and caregiver depressive symptoms and rating accuracy as well as a link between accuracy and QOL for both patients and caregivers. While often used out of necessity to assess patient symptoms when the patient is unable to self-report, we would offer caution in interpretation of proxy-reporting for symptom severity and interference in neuro-oncology settings, knowing that psychosocial function within the family may be important for accuracy of proxy report. Clinical interpretation of proxy reports may benefit from an understanding of illness communication and caregiver depressive symptoms within the reporting dyad, with an awareness that proxy reports from dyads with poor illness communication or where a caregiver reports depressive symptoms may have reduced accuracy.

## Conclusions

The psychosocial context of the family, including dyadic illness communication and depressive symptoms, plays an important role in the accuracy of symptom understanding. Inaccurately understanding patients experience is related to poor QOL for both patients and caregivers, pointing to important targets for symptom management interventions that involve family caregivers.

## Data Availability

The datasets generated and/or analyzed during the current study are not publicly available due the rareness of the disease but are available from the corresponding author on reasonable request.
